# *Crithidia fasciculata* gene of unknown function CFAC1_070014400 encodes a putative alpha/beta hydrolase with a conserved active site

**DOI:** 10.17912/micropub.biology.001967

**Published:** 2026-04-03

**Authors:** Justin Davis

**Affiliations:** 1 Department of Biology, North Carolina Wesleyan University, Rocky Mount, North Carolina, United States

## Abstract

A gene of unknown function, CFAC1_070014400 (Transcript ID: CFAC1_070014400.1), identified in *Crithidia fasciculata* was analyzed using bioinformatic methods to study the sequence and structure of its encoded protein. Results from domain predictions, conservation analysis, and structural comparisons indicate the encoded protein is a member of the alpha/beta hydrolase superfamily and contains a conserved active site. Based on phylogenetic analysis, the encoded protein groups with other putative alpha/beta hydrolase homologs from related kinetoplastid parasites.

**Figure 1. Sequence and Structural Characterization of CFAC1_070014400:** A) Domain structure of CFAC1_070014400 indicating the location of predicted sequence features. Two primary domains include a putative alpha/beta hydrolase fold and a predicted transmembrane domain. Created using Illustrator for Biological Sequences (Liu et al., 2015) based on predictions from InterPro (Blum et al., 2020). (B) Maximum likelihood phylogenetic tree calculated using the MEGA12 evolutionary analysis software using default settings (Kumar et al., 2024). Support values are shown from 500 bootstrap repetitions. (C) Multi-Sequence Alignment of the alpha/beta hydrolase fold domain showing sequence conservation across diverse homologs from related kinetoplastid parasites. The multi-sequence alignment was created using Clustal Omega then exported to Jalview for visualization (Sieviers and Higgins 2017; Waterhouse et al., 2009). Red arrows indicate the residues of the predicted catalytic triad (Ser102, His271 and Asp239), which are highly conserved and predicted to be functional. ConSurf-identified highly-conserved functional residues in CFAC1_070014400 indicated with asterisks (Ashkenazy et al., 2016). (D) AlphaFold3 (Abramson et al., 2024) predicted structural model of the full length CFAC1_070014400 protein. Dark blue represents a very high model confidence (pLDDT >90), light blue represents medium confidence (90 >pLDDT >70), yellow represents low confidence (70 >pLDDT >50), and orange represents very low confidence (pLDDT <50). (E) Structural overlay of the CFAC1_070014400 alpha/beta hydrolase domain (Blue) with alpha/beta hydrolase-1 domain-containing protein (yellow) from *Leishmania infantum* ( *L. infantum* ). Structure alignment was done using the Foldseek server and the AFDB-proteome database (Kempen et al., 2024). The side box shows the predicted active site containing the conserved catalytic triad. The bottom portion shows a model representation of the catalytic triad. (F) AlphaFold3 predicted the structural model of the CFAC1_070014400 alpha/beta hydrolase domain with coloring based on ConSurf conservation scores.

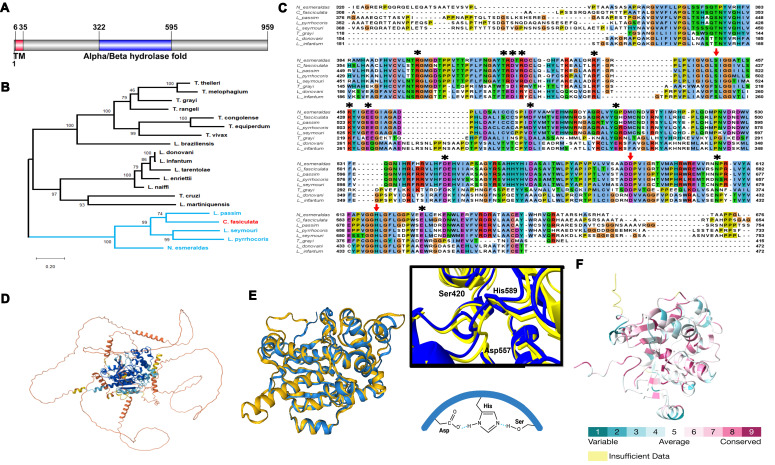



## Description

Several members of the family *Trypanosomatidae* are of importance to global health. The family *Trypanosomatidae* is composed entirely of protozoan parasites, which includes clinically relevant human parasites like *Trypanosoma brucei* ( *T. brucei* ) and *Leishmania* spp. *T. brucei* causes African sleeping sickness and members of the *Leishmania* genus causes Leishmaniasis in humans. The genomes of these parasites have been sequenced and provided novel insights into parasite biology (Berriman et al., 2005; Ivens et al., 2005). Understudied members of this family include parasites like *Crithidia fasciculata* ( *C. fasciculata* ) that complete their monoxenous life cycles in a single mosquito host. *C. fasciculata’s* genome has been sequenced and has been used as a model organism to study the related human pathogens, *Trypanosoma brucei* and *Leishmania* (Filosa et al., 2019). A vast majority of *C. fasciculata* genes are annotated as hypothetical proteins with unknown function. Here, we predict the encoded function of an unknown gene in *C. fasciculata* to advance our understanding of parasite genetics.

Based on our analysis, the uncharacterized *C. fasciculata* gene CFAC1_070014400 (Shanmugasundram et al., 2023) is a member of the alpha/beta hydrolase superfamily, subfamily 4 (IPR050960). Members of this family carry out diverse sets of enzymatic functions associated with lipid metabolism (Lenfant et al., 2013). alpha/beta hydrolase domain-containing proteins are understudied in kinetoplastid parasites, but phospholipases have been recognized for their importance in pathogenesis in related parasites *T. brucei* and *Leishmania* spp. (Bordon et al., 2018; Monic et al., 2022). Based on our analysis, CFAC1_070014400 also contains a single transmembrane helix, which suggests that it has a possible membrane localization. Other alpha/beta hydrolase domain-containing proteins, found in mammalian cells, are anchored to membranes by a single transmembrane helix and play key roles in lipid metabolism and signalling (Lord et al., 2013).

The InterPro web server (Blum et al., 2025) identified the 959-amino acid CFAC1_070014400 protein as a member of the alpha/beta hydrolase superfamily, subfamily 4 (IPR050960). The sequence was found to contain an alpha/beta hydrolase fold (IPR029058) and a single transmembrane helix ( **Figure 1A** ).&nbsp;

The softwares Phobius (Käl et al., 2004l) and TMHMM (Hallgren et al., 2022) both identified a single transmembrane helix at the N-terminus of CFAC1_070014400 (6-35) ( **Figure 1A** ). A putative signal peptide was also identified using both TargetP (Armenteros et al., 2019) and DeepLoc (Thumuluri et al., 2022). DeepLoc predicted the membrane association of CFAC1_070014400 to be peripheral (0.7540). The softwares BUSCA (Savojardo et al., 2018) and DeepLoc both predicted the CFAC1_070014400 protein to be membrane associated, but no confident prediction of subcellular localization could be determined. BUSCA predicted CFAC1_070014400 to localize to the plasma membrane, while DeepLoc predicted the localization to be the lysosome/vacuole.

We used the evolutionary analysis software MEGA12 (Kumar et al., 2024) to construct a maximum likelihood phylogenetic tree with the CFAC1_070014400 protein and putative homologs identified by an NCBI BlastP search in related kinetoplastid parasites ( **Figure 1B** ). CFAC1_070014400 appears to be most closely related with putative homologs from other monoxenous trypanosomatid parasites.&nbsp;

Using an NCBI BlastP search, homologs of the CFAC1_070014400 protein were identified across a diverse set of kinetoplastid parasites. These included monoxenous trypanosomatids, as well as *Leishmania* spp. and *Trypanosoma.* Homologs were also identified in diverse species belonging to Plantae and Fungi. No homologs were identified in Animalia, Archaebacteria, or Bacteria. We used ClustalOmega and Jalview (Sieviers and Higgins 2017; Waterhouse et al., 2009) to construct a multiple sequence alignment ( **Figure 1C** ) in order to visualize sequence conservation across representative homologs. There appears to be significant sequence conservation within the alpha/beta hydrolase domain, which indicates these residues are likely important across multiple species. Additionally, many of these residues were identified by ConSurf (Ashkenazy et al., 2016) to be highly conserved across multiple species as well as functional and are marked with asterisks. These included residues of the putative catalytic triad (Ser102, His271 and Asp239). The full ConSurf results for CFAC1_070014400 are available as extended data.&nbsp;

We used AlphaFold3 (Abramson et al., 2024) to predict a tertiary structure of the full length CFAC1_070014400 protein ( **Figure 1D** ). The program predicted confident folding in the putative Alpha/Beta hydrolase domain (residues 322- 595). The core structural region consists of beta-sheets flanked by alpha helices. This structural core is conserved and is similar to what is observed in other alpha/beta hydrolases across diverse eukaryotes (Ozhelvaci and Steczkiewicz 2025).&nbsp; When the putative alpha/beta hydrolase domain of CFAC1_070014400 was overlaid with its nearest structural homolog from *L. infantum,* we observed many features that were overlaid with each other including the putative catalytic triad ( **Figure 1E** ).&nbsp; The putative catalytic triad we identified in CFAC1_070014400 consisted of the residues, Ser102, His271 and Asp239. This canonical catalytic triad is highly conserved in alpha/beta hydrolase family proteins and generally consists of a histidine, an acid (aspartic acid or glutamate) and a nucleophilic residue (Ozhelvaci and Steczkiewicz 2025). In mammalian alpha/beta hydrolase proteins, a serine residue in the active site generally serves as the nucleophile (Lord et al., 2013). In our identified active site, a histidine is present, and aspartic acid likely acts as the acidic residue while serine would likely act as the nucleophile. Mutagenesis studies have previously shown that mutating even one of the amino acid residues in the catalytic triad, results in a complete loss of enzyme function (Navia-Paldanius et al., 2012). Our analysis also showed that residues within this putative alpha/beta hydrolase domain are highly conserved including our putative catalytic triad ( **Figure 1F** ). This highlights the key role these residues play in enzymatic activity and the likely reason they remain highly conserved across a variety of eukaryotes.&nbsp;

Based on sequence and structural features, we predict CFAC1_070014400 is part of the alpha/beta hydrolase superfamily. Additionally, based on previously published studies of&nbsp; alpha/beta hydrolase domain-containing proteins as well as structural analysis of our protein,&nbsp; CFAC1_070014400 might be involved in lipid metabolism and signaling. Lipid metabolism plays key roles in *C. fasciculata* parasites including, membrane homeostasis, metabolism and environmental stress resistance. Next steps for functional studies of this protein could involve expressing and purifying the protein in a heterologous system to perform esterase/lipase screens to confirm alpha/beta hydrolase activity and identify putative substrates. Site-directed mutagenesis could then be performed to mutate the predicted residues in the catalytic triad to confirm involvement in enzymatic activity. Taken together, these experiments would confirm the bioinformatics predictions presented here.

## Data Availability

Description: Full ConSurf results for the CFAC1_070014400 protein. . Resource Type: Text. DOI: https://doi.org/10.22002/np0r4-pwt21
